# Alemtuzumab in chronic lymphocytic leukemia

**DOI:** 10.3747/co.2007.118

**Published:** 2007-06

**Authors:** G. Fraser, C.A. Smith, K. Imrie, R. Meyer, the Hematology Disease Site Groupof Cancer Care Ontario’s Program in Evidence-Based Care

**Keywords:** Alemtuzumab, Campath, chronic lymphocytic leukemia, systematic review, clinical practice guideline

## Abstract

**Questions:**

- With respect to outcomes such as survival, response rate, response duration, time to progression, and quality of life, is alemtuzumab a beneficial treatment option for patients with B-cell chronic lymphocytic leukemia (cll)?
- What toxicities are associated with the use of alemtuzumab?
- Which patients are more likely—or less likely—to benefit from treatment with alemtuzumab?

**Perspectives:**

Evidence was selected and reviewed by one member of the Hematology Disease Site Group (dsg) of Cancer Care Ontario’s Program in Evidence-Based Care (pebc) and by methodologists. The practice guideline report was reviewed and approved by the Hema-tology dsg, which comprises hematologists, medical and radiation oncologists, and a patient representative. As part of an external review process, the report was disseminated to obtain feedback from practitioners in Ontario.

**Outcomes:**

Outcomes of interest were overall survival, quality of life, response rates and duration, and adverse event rates.

**Methodology:**

A systematic review of the medline, embase, HealthStar, cinahl, and Cochrane Library databases was conducted to search for primary articles and practice guidelines. The evidence informed the development of clinical practice recommendations. The evidence review and recommendations were appraised by a sample of practitioners from Ontario, Canada, and were modified in response to the feedback received. The systematic review and modified recommendations were approved by a review body within the pebc.

**Results:**

The literature review found no published randomized controlled trials (rcts) that evaluated alem-tuzumab alone or in combination with other chemotherapeutic agents for the treatment of relapsed or refractory cll.

One rct evaluated alemtuzumab administered to consolidate a complete or partial response to first-line fludarabine-containing chemotherapy. That study was stopped early because of excessive grades 3 and 4 infection-related toxicity in the alemtuzumab arm. Patients receiving alemtuzumab experienced significantly improved progression-free survival as compared with patients undergoing observation.

Six single-arm studies evaluated disease response with administration of alemtuzumab as a single agent in the treatment of patients with relapsed or refractory cll post-fludarabine. The pooled overall response rate was 38% (complete response: 6%; partial response: 32%). Adverse events associated with the use of alemtuzumab were commonly reported and included serious infusion-related, hematologic, and infection-related toxicities.

**Recommendation:**

This evidence-based recommendation applies to adult patients with B-cell cll.

Treatment with alemtuzumab is a reasonable option for patients with progressive and symptomatic cll that is refractory to both alkylator-based and fludarabine-based regimens.

**Qualifying Statements:**

The evidence supporting treatment with alemtuzumab comes principally from case series that evaluated disease response as the primary outcome measure. Patients should be informed that any possible beneficial effect of alemtuzumab on other outcome measures such as duration of response, quality of life, and overall survival are not supported in evidence and currently remain speculative.

Treatment with alemtuzumab is associated with significant and potentially serious treatment-related toxicities. Patients must be carefully informed of the uncertain balance between potential risks of harm and the chance for benefit reported in studies. Given the current substantial uncertainty in this balance, patient preferences will likely play a large role in determining the appropriate treatment choice.

Given the potential toxicities associated with alemtuzumab, and given the limited nature of the agent’s testing in clinical trials in broad populations of patients with cll, the use of alemtuzumab in patients with important comorbidities may be associated with excessive risks.

## 1. QUESTIONS

- With respect to outcomes such as survival, response rate, response duration, time to progression, and quality of life, is alemtuzumab a beneficial treatment option for patients with B-cell chronic lymphocytic leukemia (cll)?
- What toxicities are associated with the use of alemtuzumab?
- Which patients are more likely—or less likely—to benefit from treatment with alemtuzumab?

## 2. CHOICE OF TOPIC AND RATIONALE

With an incidence of 4 per 100,000 population, cll is the most common form of adult leukemia in the Western hemisphere. In patients older than 70 years, the incidence approaches 50 per 100,000.

Established diagnostic criteria allow cll to be differentiated from related subtypes of indolent non-Hodgkin lymphoma 1. Patients requiring therapy are usually treated either with systemic alkylator-based chemotherapy or with a purine analogue (fludara-bine). Unfortunately, cll remains incurable with conventional chemotherapeutic approaches, and patients will relapse even after a favourable response to first-line therapy.

Several randomized controlled trials (rcts) in patients with untreated, advanced-stage cll have documented superior response rates and response duration in patients randomized to fludarabine than in patients treated with alkylator-based chemotherapy 2–4. But despite those encouraging results, an improvement in overall survival has not been shown. Patients with disease refractory to standard chemotherapy have a particularly poor prognosis, and no currently accepted standard treatment exists. New therapies and treatment approaches are needed to improve outcomes for patients with cll.

Monoclonal antibodies are an emerging class of drugs with a unique mechanism of action that represents a novel approach to cancer treatment. Rituximab, a humanized anti-CD20 monoclonal antibody, has proven to be particularly effective for patients with B-cell lymphomas. Alemtuzumab, a humanized anti-CD52 monoclonal antibody, was the first of this class of drugs to receive U.S. Food and Drug Administration approval for use in the treatment of patients with cll relapsed or refractory to fludarabine. Alemtuzumab is currently under review for approval in Canada. Although the function of CD52 is not known, this antigen is expressed on a variety of hematopoietic cells, including normal and malignant T- and B-lymphocytes; CD52 is not expressed on hematopoietic stem cells. Once bound to CD52, alemtuzumab induces cell death by one or more of the following mechanisms:

- Complement-dependent cellular cytotoxicity
- Antibody-dependent cellular cytotoxicity
- Induction of apoptosis

Clinical activity has been demonstrated in heavily pretreated patients, including those with disease progression following treatment with fludarabine. However, the benefits of alemtuzumab are offset by potential toxicities, including infection-related morbidity and mortality.

Because licensing approval may precede the publication of phase iii studies, the Hematology Disease Site Group (dsg) felt that a systematic review of the current literature was needed. This systematic review will inform further recommendations on this topic when updated with relevant, high-quality evidence in the future.

## 3. METHODS

### 3.1 Review Development

The present systematic review was developed by the Hematology dsg of Cancer Care Ontario’s Program in Evidence-Based Care (pebc). Evidence was selected and reviewed by one member of the Hematology dsg.

This systematic review is a convenient and up-to-date source of the best evidence available on alemtuzumab in cll. The body of evidence in the present review primarily consists of mature rct data, where available. That evidence forms the basis of a clinical practice guideline developed by the Hema-tology dsg. The systematic review and companion practice guideline are intended to promote evidence-based practice in Ontario, Canada. The pebc is editorially independent of Cancer Care Ontario and the Ontario Ministry of Health and Long-Term Care.

### 3.2 Literature Search Strategy

A systematic search of the published literature was conducted to identify all reports relating to the use of alemtuzumab for the treatment of patients with cll. The medline (1966 to July 2005), cinahl (1982 to July 2005), HealthStar (1975 to July 2005), cancerlit (1975 to July 2005), premedline (July 2005), Cochrane Controlled Trials Register (July 2005), and Cochrane Database of Systematic Reviews (July 2005) databases were searched. In addition, the proceedings of the annual conferences of the American Society of Hematology for 1995–2004 and the Ameri-can Society of Clinical Oncology (asco) for 1995–2005 were searched for relevant abstracts. The databases of the United Kingdom Coordinating Committee on Cancer Research Register, Physician Data Query, National Institute of Health Clinical Trials, and the European Organization for Research and Treatment of Cancer were searched for ongoing clinical trials. The National Guidelines Clearinghouse was searched for clinical practice guidelines.

Only studies published in English were selected for the literature review. Publications evaluating alemtuzumab in non-human subjects and those categorized as “published comments,” “letters,” and “editorials” were excluded. The reference list from each selected article was also reviewed. Where it was deemed necessary, the authors of included publications were contacted for missing or additional data.

The preliminary literature search was performed in November 2002; that search was subsequently updated in November 2004 and July 2005. After the preliminary search, the study selection criteria were amended to exclude studies with fewer than 20 evaluable patients. As a result, studies in the preliminary literature search that had fewer than 20 evaluable patients were later removed from the report. The data from those small studies, had they been included, would not have significantly affected either the results or the dsg’s recommendations. For the sake of clarity, results from the preliminary and updated searches are presented together in the present systematic review.

### 3.3 Study Selection Criteria

Articles were selected for inclusion in this systematic review if they met the following criteria:

- The study group included patients with cll.
- The role of alemtuzumab, as a single agent or in combination with other therapy, was being tested for either induction or consolidation therapy.
- Any of the following outcomes was being reported: survival, quality of life, time to progression, response duration, response rate, or adverse effects.
- The sample size reached a minimum of 20 evaluable patients.

Two independent observers reviewed the title and abstract of each publication. These observers were blinded to author name, institution, name of journal, nature of the paper (full paper or abstract), and results. The blinded observers then scored each abstract as follows:

- “Yes” for those that met the inclusion criteria
- “No” for those that failed to meet the inclusion criteria
- “Maybe” for those about which the observer was uncertain

If both observers agreed that the abstract met the inclusion criteria, the complete document, if available, was retrieved for further analysis. In cases of disagreement, both observers reassessed the blinded abstract together to achieve consensus. Where consensus could not be reached, or in cases where both observers scored the abstract as “maybe,” the full document was retrieved and assessed by both reviewers to achieve consensus regarding eligibility. The reasons that retrieved articles were excluded are documented.

### 3.4 Synthesizing the Evidence

A lack of adequately designed rcts in the sample meant that a formal meta-analysis was deemed inappropriate. Where possible, response rates from single-arm studies evaluating similar patient groups were calculated. Data were pooled using intent-to-treat groups, and response proportions were computed.

## 4. RESULTS

### 4.1 Literature Search Results

The original and updated searches found 527 publications, with forty of those publications meeting the inclusion criteria. Of those forty citations, eighteen were subsequently excluded from analysis for these reasons:

- Duplicate publication (*n* = 1)
- Anecdotal case reports (*n* = 3: one report of severe immune thrombocytopenic purpura following a 10-week course of alemtuzumab; one report of gas gangrene 6 weeks after an 8-week course of alemtuzumab; and one report of a patient with cll treated with 3 courses of alemtuzumab over a 3-year period)
- Evaluation of patients with Sezary syndrome (*n* = 1)
- Evaluation of non-clinical outcomes (*n* = 1: reported T-cell subset recovery after treatment with alemtuzumab; the clinical outcomes were reported in a separate publication that was included in the present systematic review)
- Abstracts subsequently published as full papers (*n* = 11; all of which, as full papers, met the inclusion criteria for the present systematic review)

These twenty-two publications were eligible for review (Table I):

- Single-arm studies evaluating alemtuzumab as a single agent in patients with relapsed or refractory cll (*n* = 9: four full papers, five abstracts)
- Studies evaluating alemtuzumab as a single agent in newly diagnosed patients with previously untreated cll (*n* = 3: two full papers, one abstract; the abstract publication reported only preliminary toxicity data from an rct comparing alemtuzumab with chlorambucil as a first-line treatment in cll)
- Single-arm studies evaluating alemtuzumab in combination with additional agents for patients with refractory cll (*n* = 3: two full papers, one abstract)
- Studies evaluating alemtuzumab as consolidation therapy in cll patients with a “response” to previous-line therapy (*n* = 6: one full paper, five abstracts; the full paper reported results from an rct comparing alemtuzumab maintenance therapy with observation alone in patients with a response to first-line fludarabine, a trial that was stopped early because of severe infection-related complications in the patients randomized to the alemtuzumab arm)

The remaining citations reported results from single-arm studies. One publication reported a pooled analysis for the risk of cytomegalovirus (cmv) reactivation, cmv pneumonia, and cmv-related death in patients with lymphoid malignancies treated with alemtuzumab.

Seven published practice guidelines on the management of cll were retrieved. Two of those were excluded from the present report because they were not published in English. The European Society for Medical Oncology (esmo), the German CLL Study Group, and the Guidelines Working Group of the U.K. CLL Forum published separate guidelines for the diagnosis, staging, and treatment of patients with cll, all of which included reference to alemtuzumab therapy. One published practice guideline by Keating *et al.* (2004) 26 specifically addressed the use of alem-tuzumab in cll.

The esmo guideline lacked

- a description of the methods used to develop its recommendations;
- specific mention of the response rates, response durations, and associated toxicities found in the included studies; and
- explicit indications about which studies informed which recommendations.

The German cll guideline was described as a review article and stated that it was a consensus document of the German CLL Study Group (with membership listed). No description of the methods used to produce the guideline were provided. Two studies evaluating alemtuzumab were cited within the text of the document, and those studies were also retrieved in the literature search for the present report (one study was excluded because of the sample size criterion). Definitive recommendations regarding the use of alemtuzumab in cll were not provided in the German publication.

The U.K. CLL Forum guideline described the methods used to develop the recommendations and explicitly indicated which studies informed the various recommendations. Outcomes data, including response rates, duration of response, and median survival rates observed in trials were reported. Nine single-arm studies of alemtuzumab in patients with cll informed that guideline. Of those studies, six are included in our report, and three were excluded because they did not meet the minimum sample size criterion.

The practice guideline that specifically addressed alemtuzumab use indicated that it was developed out of an expert-opinion roundtable on the topic held August 8–9, 2004). No further description of the methods used was provided. The Keating *et al.* guideline 26 was informed by evidence from eight trials of alemtuzumab in cll, all of which are included in the present report.

The recommendations of the foregoing practice guidelines, which concern alemtuzumab use in patients with cll, are addressed here in the Discussion section.

### 4.2 Outcomes

#### 4.2.1 Question 1

*With respect to outcomes such as survival, response rate, response duration, time to progression, and quality of life, is alemtuzumab a beneficial treatment option for patients with B-cell chronic lymphocytic leukemia (cll)?*

No studies reported quality-of-life outcome data.

##### Single-Agent Alemtuzumab for Relapsed/Refractory cll

###### Response Rates

The overall response (or), complete response (cr), and partial response (pr) rates associated with single-agent alemtuzumab for patients with relapsed or refractory cll are summarized in Table II and include data from nine single-arm studies. No comparative or randomized studies were available for analysis. Six trials each evaluated a standard 12-week course of alemtuzumab in patients with relapsed or refractory disease post therapy with fludarabine 5,6,9,10,12,13. The combined or rate across those six trials was 38% (range: 31%–41%), and the combined cr and pr rates were 6% (range: 1%–10%) and 32% (range: 26%–38%) respectively. One study 10 evaluated alemtuzumab administered subcutaneously and reported or and cr rates similar to those seen in studies with intravenous administration. No trials directly compared subcutaneous with intravenous administration.

Three studies administered alemtuzumab for longer than 12 weeks. A single-arm study by Moreton *et al.* 11 evaluated treatment with alemtuzumab until a maximal clinical response was achieved in patients with relapsed or refractory disease post therapy with fludarabine. Rates of 54%, 35%, and 19% for or, cr, and pr respectively were reported for 91 patients treated for a median of 9 weeks (range: 1–16 weeks). Peripheral blood and bone marrow samples were obtained from all patients before, during, and after alemtuzumab therapy to evaluate minimal residual disease (mrd) status. A highly sensitive and validated four-colour flow cytometry–based assay was used to define mrd status. The limit of detection for that assay was approximately one cll cell in 10^4^–10^5^ leukocytes 27. In 20% of patients, an mrd-negative remission was achieved in the bone marrow and peripheral blood. However, those patients had a median treatment-free period, before initiation with alemtuzumab, of 10 months (range: 4–43 months), and most patients (72%) had no evidence of lymphadenopathy or splenomegaly before alemtuzumab treatment. No trials have directly compared different alemtuzumab regimens.

The remaining two studies 7,8 administered therapy to 16 and 30 weeks respectively, had smaller sample sizes (24 and 27 patients respectively), and reported response rates similar to those of the other studies in the group.

###### Response Duration

Data on median time to progression (ttp) were reported in five single-arm studies evaluating alemtuzumab in patients with disease that had relapsed after, or was refractory to, fludarabine ) therapy (Table II 5–7,10,11. Fludarabine-refractory disease was usually defined as either no response to fludarabine or relapse within 6 months following a response to fludarabine. The median ttp ranged from 4 months to 10 months.

Moreton *et al.* 11 compared the median treatment-free survival (tfs) according to the response to alemtuzumab (mrd-negative cr, mrd-positive cr, pr, or no response). Patients achieving mrd-negative cr had a significantly prolonged tfs as compared with those having an mrd-positive cr, a pr, or no response (median tfs not reached: 20 months, 13 months, and 6 months respectively; *p* < 0.0001). The median tfs for the entire cohort was not reported.

###### Survival

Survival data were reported in four single-arm studies evaluating alemtuzumab in patients with relapsed or refractory disease post fludarabine ) (Table II 5–7,10. Median overall survival (os) ranged from 8 months to more than 2 years.

Moreton *et al.* 11 compared os according to response to alemtuzumab. Patients achieving an mrd-negative cr had a significantly prolonged os as compared with those having an mrd-positive cr, a pr, or no response (median os not reached: 60 months, 70 months, and 15 months respectively; *p* < 0.0007). Median os for the entire cohort was not reported.

##### Single-Agent Alemtuzumab for Previously Untreated cll

###### Response Rates

Two studies investigated the or, cr, and pr rates associated with a trial of single-agent alemtuzumab for patients with previously untreated cll 15,16. Lundin *et al.* 15 reported an or rate of 87% for 38 evaluable patients treated with subcutaneous alemtuzumab for 18 weeks; the cr and pr rates were 19% and 68% respectively. Most patients had advanced-stage disease (69% Rai III/IV).

###### Response Duration

In the trial by Lundin *et al.* 15, median time to treatment failure (ttf) had not been reached at 18 months. In an update of that trial, reported in abstract form, median ttf in responders had not been reached at 35 months 16. No other trials reported data pertaining to response duration.

###### Survival

No studies reported os rates associated with alemtuzumab therapy for previously untreated patients with cll.

##### Alemtuzumab in Combination with Additional Agents for Relapsed/Refractory cll

###### Response Rates

Three single-arm studies evaluated alemtuzumab-containing combination regimens for the treatment of relapsed or refractory cll (Table II) 17–19. No trials directly compared different combination regimens. One trial, Elter *et al.* 19, evaluated alemtuzumab in combination with fludarabine and reported an or rate of 83% for 36 evaluable patients. The cr and pr rates were 31% and 53% respectively.

Faderl *et al.* 17 reported a 63% or rate (6% cr 57% pr) for 32 patients treated with alemtuzumab in combination with rituximab. Wierda *et al.* 18 evaluated a regimen consisting of cyclophosphamide, fludarabine, alemtuzumab, and rituximab administered over six 28-day cycles; the overall response rate was 52% (14% cr, 38% pr).

###### Response Duration

Elter *et al.* 19 reported a median ttp of 13.0 months for the entire patient cohort; for patients who achieved a cr, median ttp was 21.9 months. No other studies reported data for response duration associated with alemtuzumab-containing combination regimens for patients with relapsed or refractory cll.

###### Survival

Elter *et al.* 19 reported a median os of 35.6 months. For patients who achieved cr, median os was not reached. No other studies reported survival data.

##### Alemtuzumab Consolidation for Patients with a Response to Previous-Line Therapy

###### Response Rates

One rct^20^ and four single-arm studies^21^–^24^ reported response rates for alemtuzumab consolidation therapy; Table III summarizes the results. The German CLL Study Group (Wendtner *et al.*) published results from an open-label, multicentre, randomized phase III trial that compared 12 weeks of alemtuzumab consolidation with observation in patients achieving at least a pr after 6 cycles of first-line fludarabine-containing chemotherapy 20. The study’s sample size of 90 patients was designed to have an 80% statistical power to detect a 25% improvement in progression-free survival (pfs) at 2 years. The trial was stopped after the accrual of 21 patients because of the occurrence of grades 3 and 4 infections (National Cancer Institute Common Toxicity Criteria, version 2.0) in 7 of the first 11 patients randomized to alemtuzumab consolidation. Of those 11 patients, 2 (18%) improved on their response to first-line therapy; both patients achieved a pr following first-line fludarabine-containing chemotherapy and improved to a cr following consolidation with alemtuzumab.

The four single-arm studies evaluating alem-tuzumab consolidation therapy were reported in abstract form only 21–24. All studies evaluated a 4- to 8-week course of alemtuzumab in patients who had stable disease or better after first- or second-line chemotherapy. Response to alemtuzumab consolidation was generally defined as an improvement in “post-induction” response status according to National Cancer Institute Working Group criteria. Overall, response status improved following alemtuzumab consolidation. Two studies 21,23 documented an mrd-negative remission status in 38%–51% of patients, based on clonality of the immunoglobulin H (IgH) gene rearrangement by polymerase chain reaction analysis of samples of peripheral blood or bone marrow, or both.

###### Response Duration

Two studies reported data for response duration associated with alemtuzumab consolidation following a response to first- or second-line chemotherapy 20,23. In the rct published by the German CLL Study Group 20, no progression occurred in the 11 patients randomized to alemtuzumab consolidation; that results compares with a 24.7-month mean pfs in the 10 patients randomized to observation (*p* = 0.036). O’Brien *et al.* 23 reported a median ttp of more than 24 months in patients who demonstrated a response to alemtuzumab consolidation.

###### Survival

Survival data associated with the use of alemtuzumab consolidation therapy were reported in the rct published by the German CLL Study Group 20 Median os had not been reached in either the alemtuzumab arm or the observation arm. No other studies reported survival data.

#### 4.2.2 Question 2

What toxicities are associated with the use of alemtuzumab?

Toxicities associated with the administration of alemtuzumab were reported in most studies (Table IV). The most common adverse events can be broadly grouped into these categories

- Infusion-related side effects
- Myelosuppression
- Infection-related toxicities

##### Infusion-related side effects

Infusion-related side effects were reported in sixteen studies 5–7,9,11,12,14–20,22–24. They occurred in most patients treated with intravenous alemtuzumab, were usually grade 1 or 2 in severity, and were manageable with appropriate supportive care. The prophylactic use of pre-medications was reported in about one third of the studies and usually consisted of orally administered acetami-nophen and antihistamines; corticosteroids were generally reserved for more severe reactions. Grade 3 or 4 fever, rigour, and nausea were reported in up to 20% of patients; other serious infusion-related toxicities were less common. The incidence of infusion-related side effects was similar regardless of the population evaluated, tended to be higher and more severe with the first infusion, and improved with subsequent courses of treatment.

The subcutaneous administration of alemtuzumab was reported in three trials 10,15,21, and this route was generally much better tolerated than the intravenous route used in similar patients (Table IV). Grade 1 or 2 fever (68%) and local injection site reactions (88%) were reported; grade 3 or 4 reactions of any kind were rarely reported (fewer than 2% of patients) 15.

##### Myelosuppression

Data regarding myelosuppression associated with the use of alemtuzumab were reported in 10 trials 6,7,9–12,15,18–20. Results for studies evaluating various disease populations were analyzed separately.

Grades 3 and 4 myelosuppression were common in studies evaluating alemtuzumab monotherapy for patients with relapsed or refractory disease 6,7,9–12. The pooled estimates for grades 3 and 4 neutropenia, thrombocytopenia, and anemia were 39% (range: 22%–66%), 31% (range: 23%–46%), and 8% (range: 0%–28%) respectively. Similar rates of grades 3 and 4 myelosuppression were reported for studies evaluating alemtuzumab in combination and in maintenance or consolidation regimens. Data regarding the co-administration of hematopoietic growth factors were not well reported.

##### Infection-Related Toxicity

Data regarding the incidence of infections in patients treated with alem-tuzumab were reported in twenty publications 5–20,22,23, 25,28. In thirteen studies, antimicrobial prophylaxis was administered during alemtuzumab therapy. The most frequently cited combination was cotrimoxazole together with antiviral therapy (acyclovir, valacyclovir, famciclovir) for the prevention of *Pneumocystis carinii* pneumonia (pcp) and herpes virus infections. For the present systematic review, data relating to infection-related toxicity were analyzed and reported separately for various study populations.

###### Single-Agent Alemtuzumab for Relapsed or Refractory cll

Data pertaining to infection-related morbidity in patients with relapsed or refractory cll were reported in eight studies 5–12. The per capita incidence of all infections ranged from 30 to 93 per 100 patients (46 per 100 patients across studies). The incidence of grades 3 and 4 infections ranged from 7 to 36 per 100 patients (18 per 100 across studies), and infection-related mortality ranged from 0 to 10 per 100 patients (4.5 per 100 across studies).

Grades 3 and 4 infections included disseminated viral infections [for example, varicella–zoster virus and herpes simplex virus (hsv)], systemic *Candida* infections, mycobacterial reactivation, and invasive fungal infections (for example, pulmonary aspergillo-sis, rhinocerebral mucormycosis, and cryptococcal meningitis and pneumonia). Infection with pcp was reported, but these cases generally occurred in patients not receiving prophylaxis.

The incidence of cmv reactivation was reported in seven of the above-mentioned trials 5,6,8–11,13 and ranged from 1% to 29% (9% across studies); cmv pneumonitis was reported in 4 patients (0.8% across studies). The actual risk of cmv reactivation in this patient population was not clear because most studies did not prospectively screen all patients.

Williams *et al.* 28 retrospectively pooled safety data in 1538 patients with lymphoid malignancies treated with alemtuzumab in five single-arm trials and reported that 3.6% of patients experienced “symptomatic” cmv reactivation, cmv pneumonitis (0.8%), and cmv-related death (0.2%). Routine prospective screening of all patients for cmv reactivation was not performed in those studies. Patients who developed cmv reactivation were usually treated with intravenous ganciclovir until evidence of viremia resolution. Ganciclovir therapy was highly effective for treating cmv reactivation, but because ganciclovir-induced neutropenia was common, myeloid growth factors (for example, granulocyte-colony stimulating factor) were often co-administered.

Rates of adverse events ranged from 11% to 82% in the studies. Overall, alemtuzumab therapy was prematurely discontinued in approximately 20% of patients because of an adverse event—most often infection-related complications or myelosuppression, or both.

###### Single-Agent Alemtuzumab for Previously Untreated cll

In an rct comparing alemtuzumab to chlorambucil , Hillmen *et al.* 14 for newly diagnosed patients with cll reported a cmv reactivation rate of 15% for all patients randomized to receive alemtuzumab. All patients with detectable cmv reactivation were treated with gan-ciclovir; no cases of cmv pneumonitis occurred. Other infection-related toxicities have not yet been reported.

Lundin *et al.* 15 reported cmv reactivation in 4 patients (11%) treated with subcutaneous alemtuzumab. One case of pcp occurred in a patient not receiving prophylaxis. An update describing the long-term follow-up for that patient cohort documented 1 episode of symptomatic Epstein–Barr virus (infection 21 months post alemtuzumab therapy) 16. No other serious infections occurred.

###### Alemtuzumab in Combination with Additional Agents for Relapsed or Refractory cll

Faderl *et al.*^17^ documented infections in 52% of patients with lymphoid malignancies treated with alemtuzumab in combination with rituximab; cmv reactivation occurred in 27%. Infections in cll patients were not reported separately.

Elter 19 reported data on 36 patients treated with alemtuzumab in combination with fludarabine; fungal pneumonia (*n* = 2), cmv reactivation (*n* = 2), and infection-related death (*n* = 1 case of *Escherichia coli* sepsis) were the only reported infection-related complications.

Wierda *et al.* 18 reported cmv reactivation in 24% of patients (*n* = 21) treated with alemtuzumab in combination with cyclophosphamide, rituximab, and fludarabine.

###### Alemtuzumab Consolidation for Patients with a Response to Previous-Line Therapy

Wendtner *et al.* 20 randomized patients with a response to first-line fludarabine-containing chemotherapy to consolidation with alemtuzumab (30 mg intravenously 3 times weekly for 12 weeks) or observation. Explicit stopping rules were determined *a priori* and included grade 3 or 4 infection occurring in 5 of the first 10 patients accrued to the alemtuzumab arm. The study was stopped early because of severe infections in 7 of 11 patients randomized to alemtuzumab consolidation. Grades 3 and 4 infections included cmv reactivation (*n* = 2), cmv pneumonitis (*n* = 2), pulmonary aspergillosis and hsv/human herpes virus 6 (*n* = 1), pulmonary tuberculosis (*n* = 1), and herpes zoster reactivation (*n* = 1). An additional 2 patients developed grade 2 cmv reactivation. Overall, 9 of 11 patients (82%) randomized to alemtuzumab consolidation discontinued therapy because of an adverse event (severe infection in 5 patients and severe myelosuppression in 4 patients).

Four additional single-arm studies reported infection-related toxicity for alemtuzumab consolidation therapy 22–25. Reactivation of cmv was common, occurring in 21%–57% of patients; the single reported case of cmv pneumonitis^22^ contributed to patient death. The studies evaluated either a 10-mg or 30-mg dose of alemtuzumab administered over 6 to 8 weeks. No apparent difference in the rate or severity of infections by treatment regimen was observed.

#### 4.2.3 Question 3

Which patients are more likely—or less likely—to benefit from treatment with alemtuzumab?

Statistical evaluations for independent predictors of response, response duration, or survival were not reported in any study—including in the present systematic review. However, several publications reported subgroup analyses and clinical observations for patients who were more or less likely to respond to alemtuzumab.

Several authors noted that patients with lymphad-enopathy, particularly bulky lymph nodes (>5 cm), were less likely to achieve a clinical response to alemtuzumab-containing therapy 5,7,11,12,15,20,23. Keating *et al.* 5 reported that patients less likely to respond included those with Rai stage IV disease, with at least 1 lymph node greater than 5 cm in diameter, or with a World Health Organization (who) performance status of 2. Moreton *et al.* 11 evaluated alem-tuzumab monotherapy administered to maximal response in patients relapsed or refractory to fludar-abine and reported that patients were significantly less likely to respond if their lymph nodes were larger than 5 cm (*p* < 0.0001), if they had received 3 or more previous lines of therapy (*p* = 0.0005), or if their pre-treatment who performance status was greater than 1.

The rct published by the German CLL Study Group 20 failed to find a correlation between response status and age, disease stage, response to previous-line fludarabine-containing chemotherapy, cumulative alemtuzumab dose, duration of alemtuzumab therapy, IgH mutational status, or cytogenetic aberrations. However, their analysis was limited to just 11 patients, because the trial was stopped early because of excessive severe infections in the alem-tuzumab-consolidation arm.

## 5. DISCUSSION

In its deliberations, the Hematology dsg places particular emphasis on

- results from published rcts (where available);
- recognition of a hierarchy of outcomes that should influence treatment decisions, with priority given to therapies found to extend life or improve quality of life; and
- the potential toxicities associated with treatment, with particular emphasis on the toxicities seen in the patients most likely to make up the population eventually to be treated.

The members of the Hematology dsg had considerable difficulty reaching consensus on the appropriate wording of the recommendation for a potential indication for alemtuzumab in patients with cll. The recommendation went through multiple iterations (see Section 6.4). Based on their review of the available evidence, the dsg considered several interpretations for the use of alemtuzumab in patients with cll.

The dsg regards alemtuzumab to be an active agent for the treatment of patients with relapsed or chemotherapy-refractory CLL. That conclusion is based on response data from single-arm studies that report a pr in approximately one third of patients (recognizing that cr are uncommon). From the perspective of drug or multi-agent regimen development, these data are extremely promising and warrant further testing of alemtuzumab.

In their deliberations, the dsg cited these factors as leading to the current recommendation on alemtuzumab:

- Lack of data from properly designed rcts
- A paucity of data suggesting improved response duration, quality of life, or improved overall survival when alemtuzumab is compared with alternative treatment approaches
- Significant potential toxicity, particularly infection-related morbidity and mortality

Given the anticipated toxicity, data from rcts demonstrating improvement in clinically meaningful outcome measures—for example, time to progression, quality of life, or overall survival—are required before recommendations permitting the routine use of alemtuzumab in this patient population can be made.

The practice guidelines published by esmo 29 and the U.K. CLL Forum 30 made recommendations regarding the use of alemtuzumab in previously treated patients. The esmo guideline recommends alem-tuzumab as an option for patients with refractory disease following first-line therapy, based on the lowest-level evidence (asco level V: small case-series). In addition, the U.K. CLL Forum guideline recommends alemtuzumab for use in patients without bulky lymphadenopathy (<5 cm) who have been previously treated with alkylating agents and who are refractory to fludarabine. The evidence informing the U.K. CLL Forum recommendation was similar to the evidence contained in the present report and comprised data from a smaller selection of single-arm studies.

The German CLL Study Group determined that definitive recommendations could not be made regarding alemtuzumab use and indicated that further testing in clinical trials would be preferred 31.

Keating *et al.* 26 did not make explicit recommendations regarding the appropriateness of alemtuzumab use in cll patients, but implied that alemtuzumab is appropriate in fludarabine-refractory patients. Those authors also stated that advanced age should not be a contraindication to alemtuzumab use.

The Hematology dsg considered the above recommendations to be based on low levels of evidence and, initially, dsg members were not convinced that these recommendations could inform best clinical practice. Instead, the dsg initially concluded that potential benefits (response rates in a minority of patients; uncertain benefit in terms of response duration, overall survival, and quality of life) were offset by the potential for significant toxicity. Therefore, an initial recommendation was developed to indicate that the data were insufficient to support the routine use of alemtuzumab in patients with cll. The dsg acknowledged the potential controversy that could result from issuing a “non-permissive” recommendation regarding alemtuzumab use and the potential implications that such a recommendation might have for drug availability. The dsg was aware that its recommendations differed from those of other existing practice recommendations, including those published by esmo and the U.K. CLL Forum.

The dsg was also aware that, within the response data described in the literature reviewed, responses reached a magnitude that reporting authors—and members of the dsg—considered to be clinically important. Although the precise frequency of the responses was uncertain (and the best estimate was that they would be infrequent), the dsg acknowledged that an opportunity for such a response, even with substantial risks of toxicity, may be highly desired by some patients. The dsg attempted to reflect this sentiment by indicating that, after balancing the benefits and risks of treatment, certain patients may wish to consider a trial of therapy.

The dsg members had concerns with issuing an unclear and potentially conflicting set of recommendations, but they initially considered this option to represent the best available alternative, and they therefore offered this guidance: For patients with cll, the evidence is insufficient to recommend the use of alemtuzumab outside of clinical trials. The dsg recognizes that, in highly selected cases, after thorough consideration of the risks and benefits, a trial of alemtuzumab might be considered.

Section 6 details the subsequent practitioner feedback, and it notes that responding clinicians were generally in agreement with the synthesis and interpretation of the available literature and the resulting recommendations. However, a small number of respondents commented on the lack of clarity associated with the recommendations. As a result, the dsg members continued the consensus process in an effort to develop a clearer statement, and the dsg subsequently issued a new set of recommendations. The redeveloped recommendations state that “treatment with alemtuzumab is a reasonable option for patients with progressive and symptomatic cll that is refractory to both alkylator-based and fludarabine-based regimens.” To account for the continued concern about the level of evidence supporting this recommendation and the potential risk–benefit profile of the therapy, a detailed set of qualifying statements was also developed.

## 6. EXTERNAL REVIEW

The systematic review and practice guideline recommendations were distributed to practitioners in On-tario, Canada, for review and feedback in accordance with the practice guidelines development cycle 32,33.

### 6.1 Methods

A sample of 95 hematologists in Ontario received the survey, which consisted of items evaluating the methods, results, and interpretive summary used to inform the draft recommendations and asking whether the draft recommendations should be approved as a practice guideline. Written comments were invited. The practitioner feedback survey was mailed April 13, 2006, and a complete repeat mailing was sent thereafter. The Hematology dsg reviewed the results of the survey.

### 6.2 Results

A total of 63 responses were received from among the 95 questionnaires mailed, for a response rate of 66%. Of the 63 respondents, 30 (48%) indicated that they cared for patients for whom the guideline is relevant, and they completed the survey.

Overall, respondents showed strong support for the guideline. For questions that addressed issues such as the rationale for the guideline, the quality of the guideline, and the clarity of the recommendations, a substantial majority of respondents (87%–100%) expressed modest to “strong” support for the report (1 or 2 on a scale of 1–5, with 1 being “strongly agree,” 3 being “neither agree or disagree,” and 5 being “strongly disagree”).

With respect to the appropriateness of the recommendations, a majority of respondents agreed with the draft recommendations as stated (70%) and with their appropriateness for the target population (73%). Some respondents (20%) felt that the recommendations were excessively rigid and could not be applied to individual patients.

Respondents varied in their views regarding the clinical utility of the recommendations. Approximately half responded ambivalently when asked if the recommendations would produce more benefit than harm. Responses on whether the recommendations provided options that would be acceptable to patients varied widely (31% agreed, 38% were ambivalent, and 31% disagreed). Most respondents (69%) replied ambivalently when asked if the effect of the recommendations on patient outcomes would be obvious.

When asked to compare these recommendations with current practice, approximately half of the respondents felt that the questions were not applicable. More than half of the respondents (57%) would be comfortable with their patients receiving the care recommended in the draft document, and a sizable majority (70%) felt that the draft report should be approved as a practice guideline.

Most respondents felt that implementing the recommendations would require no reorganization of their practice, nor would it be technically challenging or expensive (57%–67%). About half of the respondents felt that the recommendations would be supported by a majority of their colleagues (52%), but many responded ambivalently to that question (34%).

A strong majority of respondents (79%) indicated that they would use the guideline in their own practice and would apply it to their patients (83%).

### 6.3 Summary of Written Comments

The main points contained in the written comments were these:

- Two respondents felt that the drug should be made available to select patients. One respondent felt that alemtuzumab should be recommended for use in patients with cll who are resistant to fludarabine-containing combination regiments with marrow infiltration as a primary treatment indication. This respondent noted that a response rate of 38% was observed in that subpopulation in a phase II trial, and that treatment options for such patients are extremely limited.
- Two respondents commented that the wording of the recommendation was unusual. One suggested that specific criteria be given for the highly specific circumstances mentioned in the recommendation.
- Two respondents agreed that the current recommendation was appropriate and that alemtuzumab should be used only in a clinical trial situation.

### 6.4 Modifications/Actions

The Hematology dsg reviewed and discussed the comments resulting from the practitioner feedback survey and addressed the written feedback as follows:

- In their deliberations, the members of the dsg were unanimous in the view that the data included in the evidence summary were generally of low methodologic quality and were characterized by a lack of prospective comparative trials, thereby precluding the development of a definitive recommendation to use alemtuzumab in patients with cll. However, the dsg acknowledged that there may be instances in which patients and physicians who are well informed of the risks and uncertain net clinical benefit might prefer treatment with alemtuzumab. In addition, individual members of the dsg shared anecdotal experiences involving carefully selected patients who derived benefit from treatment with alemtuzumab. The dsg is fully aware that anecdotal clinical experience is not a basis for informing a guideline recommendation, but the group acknowledged that such experience is consistent with available data and contributes to the general support of alemtuzumab as a reasonable option for select patients who may have few available alternatives.
- In their deliberations, the dsg members acknowledged that the current wording of the draft recommendation might be viewed as contradictory and should be revised.
- In their deliberations, the members of the dsg felt strongly that alemtuzumab is an active agent in cll and that it merits continued testing in well-designed clinical trials. However, the dsg felt that a recommendation for the use of alemtuzumab only in the setting of a clinical trial was too restrictive and did not take into consideration clinician or patient preferences to use alemtuzumab in selected circumstances.

### 6.5 Report Approval Panel Feedback

The final evidence-based report was reviewed and approved by Report Approval Panel (rap) of the pebc in April 2006. The panel normally consists of two members, including an oncologist with expertise in clinical and methodologic issues. However, in this case, the oncologist member did not participate in the rap review process because that individual was one of the authors of the report. No significant issues were raised by the other panel member, and the report was approved for distribution.

### 6.6 Additional Deliberations

The dsg discussed the practitioner feedback and again reviewed the draft recommendation at its bi-annual meeting of May 16, 2006. Feedback for the report was uniformly positive for questions related to the report development process. In contrast, feedback relating to several aspects of clinical care were generally less positive. Some respondents had noted that the initial draft recommendation could be perceived as contradictory in nature. Given those concerns, the members of the dsg felt that the draft recommendation required revision.

Following a detailed discussion, the dsg reached consensus on a revised recommendation and issued the three qualifying statements.

The dsg members remained unanimous in their view that the data for use of alemtuzumab in cll are limited and of low methodologic quality. The decision to revise the draft recommendations was therefore not attributable to an alternate interpretation of the available data. Instead, the major basis for revision were these:

- Appreciation by the dsg members that, despite no clear evidence for the inducement of durable periods of disease control or improvements in quality of life or overall survival, patients or clinicians or both may prefer to use alemtuzumab in selected instances. Inherent in this decision is an understanding that the potential risks could be substantial and the potential for benefit uncertain.
- The notion that some patients with few available treatment alternatives may derive benefit from treatment with alemtuzumab. The potential benefit was supported anecdotally by members of the dsg who cited specific examples of carefully selected patients who derived benefit following treatment with alemtuzumab.

In summary, the dsg reframed the recommendation to consider alemtuzumab to be a potential option for patients whose cll is refractory to current standard options (alkylator-based therapy and fludarabine). The limitations and risks of the alemtuzumab option are addressed in a series of qualifying statements.

## Figures and Tables

**TABLE I:** Characteristics of cited trials in chronic lymphocytic leukemia (cll)

*Regimen type*	*cllpopulation*	*Trials*		*Publication type*	
		*(*n*)*	*Design*	*Full*	*Abstract*
Monotherapy	Relapsed/refractory	9	Single-arm	Keating *et al.* 2002 5 Rai *et al.* 2002 7 Ferrajoli *et al.* 2003 9 Moreton *et al.* 2005 11	Rai *et al.* 2001 6 Fiegl *et al.* 2003 8 Stilgenbauer *et al.* 2004 10 Osterborg *et al.* 1997 12 Osuji *et al.* 2004 13
	Previously untreated	1	rct		Hillman *et al.* 2004 14,a
		2	Single-arm	Lundin *et al.* 2002 15 Karlsson *et al.* 2005 16	
Combination therapy	Relapsed/refractory	3	Single-arm	Faderl *et al.* 2003 17 Elter *et al.* 2005 19	Wierda *et al.* 2004 18
Consolidation therapy	Response to prior line	1	rct	Wendtner *et al.* 2003^20^,^b^	
		5	Single-arm		Montillo *et al.* 2004 21 Rai *et al.* 2002 22 O’Brien *et al.* 2003 23 Liggett *et al.* 2005 24 Rossi *et al.* 2004 25

a Reports preliminary toxicity data from an rct comparing alemtuzumab with chlorambucil for first-line treatment of cll (response data not yet reported).

b Trial stopped early because of excessive infection-related toxicity in patients randomized to alemtuzumab.

rct = randomized controlled trial.

**TABLE II:** Responses to monotherapy and combination therapy: single-arm trials of alemtuzumab for B-cell chronic lymphocytic leukemia (cll)

*Reference*	*Intervention*^a^	*Patients*	*Response (%)*			*ttp(months)*		*os(months)*	
		*[*N *(*n*)]*^b^	*or*	*cr*	*pr*	*All*	*crpts.*	*All*	*crpts.*
Monotherapy in relapsed/refractory cll									
Keating *et al.* 2002 5 (abs.)	Alemtuzumab	93	33	2	31	4.7	9.5	16	32
Rai *et al.* 2001 6 (abs.)	Alemtuzumab c	136	40	7	32	3.9	7.3	7.6	13.4
Rai *et al.* 2002 7	Alemtuzumab for 16 weeks c	24	33	0	33	7.1	19.6	27.5	35.8
Fiegl *et al.* 2003^8^	Alemtuzumab for 30 weeks maximum	27	41	4	37	nr	nr	nr	nr
Ferrajoli *et al.* 2003 9	Alemtuzumab	42	31	5	26	nr	nr	nr	nr
Stilgenbauer *et al.* 2004 10 (abs.)	Subcutaneous administration	50 (44)	36	2	34	9.7	nr	13.1	nr
Moreton *et al.* 2005 11	Alemtuzumab to maximum response	91	54	35	19	nr	20^d^	nr	41^d^
Osterborg *et al.* 1997 12	Alemtuzumab	29	41	4	38	nr	nr	nr	nr
Osuji *et al.* 2004 13 (abs.)	Alemtuzumab^e^	26 (23)	52	22	30	nr	nr	nr	nr
Monotherapy in previously untreated disease									
Hillmen *et al.* 2004 14 (abs.)	Alemtuzumab	149	nr	nr	nr	nr	nr	nr	nr
Lundin *et al.* 2002 15									
Karlsson *et al.* 2005 16 (abs.)	Alemtuzumab administered subcutaneously for 18 weeks vs. chlorambucil	41 (38) 148	87 nr	19 nr	68 nr	18+ nr	35+ nr	nr nr	nr nr
Combination therapy in relapsed/refractory cll									
Faderl *et al.* 2003 17	Alemtuzumab + rituximab^f^	32	63	6	56	nr	nr	nr	nr
Wierda *et al.* 2004 18 (abs.)	cfar	31 (21)	52	14	38	nr	nr	nr	nr
Elter *et al.* 2005 19	Alemtuzumab + fludarabine	36	83	31	53	13	22	36	Not reached

a Unless otherwise indicated, the intervention was alemtuzumab 30 mg administered intravenously 3 times weekly for 12 weeks.

b *N* represents the patient population; *n* is the evaluable patients, if fewer than the patient population.

c Rai 4-stage system.

d Complete remission not reached in patients negative for minimal residual disease. Numbers are for patients positive for minimal residual disease, with complete remission.

e Regimen details not reported.

f Alemtuzumab administered intravenously twice weekly for up to 8 weeks, plus rituximab (375 mg/m^2^) administered weekly for 4 weeks. or = overall response rate; cr = complete response; pr = partial response; ttp = time to progression; pts. = patients; os = overall survival; abs. = abstract; nr = not reported; cfar = cyclophosphamide 250 mg/m^2^ days 3–5, fludarabine 25 mg/m^2^ days 3–5, alemtuzumab 30 mg days 1, 3, 5, rituximab 375–500 mg/m^2^ day 2.

**TABLE III:** Responses to maintenance or consolidation therapy (or both): randomized and single-arm trials of alemtuzumab for chronic lymphocytic leukemia (cll)

*Reference*	*cllpopulation*	*Intervention*^a^	*Patients [*N *(*n*)]*^b^	*Post-induction*		*Post-alemtuzumab*		*Median*	*Median*
				*or*	*cr*	*or*	*cr*	*ttp(mos.)*	*os(mos.)*
rcts of alemtuzumab consolidation following first-line chemotherapy									
Wendtner *et al.* 2004^20^	pr or better post first-line fludarabine, fludarabine + cyclophosphamide	Alemtuzumab vs. observation	11 10	100 100	9 20	100 70	27 20	Not reached 24.7^c^	Not reached Not reached
Single-arm studies of alemtuzumab maintenance/consolidation therapy in patients with cll									
Montillo *et al.* 2004^21^ (abs.)	pr or better post first-line fludarabine	Alemtuzumab 10 mg subcutaneously, 6 weeks	35	100	29	100	83	nr	nr
Rai *et al.* 2002^22^ (abs.)	sd or better post first-line fludarabine	Alemtuzumab 6 weeks	56 (36)	55	4	92	27 (42)	nr	nr
O’Brien *et al.* 2003^23^ (abs.)	pr or better post chemotherapy	Alemtuzumab 10–30 mg, 4–8 weeks	58 (49)	100	12	100	28 (33)	24+ in resp.	nr
Liggett *et al.* 2005^24^ (abs.)	Response post first-line fludarabine + rituximab	Alemtuzumab, 4 weeks	29 (21)	100	nr	83	34	nr	nr

a Unless indicated otherwise, intervention was alemtuzumab 30 mg administered intravenously 3 times weekly for 12 weeks.

b *N* is the patient population assigned a treatment at the start of the trial; *n* is the evaluable patients at follow-up, if fewer than the patient population.

c *p* = 0.036.

or = response rate; cr = complete response; ttp = time to progression; mos. = median months; os = overall survival; rct = randomized controlled trial; pr = partial response; abs. = abstract; nr = not reported; sd = stable disease; resp. = responders.

**TABLE IV:** Toxicities associated with alemtuzumab for chronic lymphocytic leukemia (cll)

*Reference*	*Patients (*n*)*	*Prophylactic antimicrobials*	*Infusion-related*^a^	*Cytopenia, grade 3/4 (%)*^b^		*Infections*	*cmv*
				*Neutropenia*	*Thrombocytopenia*	*[% (grade 3/4 %)]*	*[% (disease %)]*
Monotherapy in relapsed/refractory cll							
Keating *et al.* 2002^5^	93	Famciclovir, tmp/smx	≤ 20	nr	nr	55 (27)	8 (0)
Rai *et al.* 2001^6^ (abs.)	136	Famciclovir, tmp/smx	nr^c^	22	23	32 (nr)	1.5 (nr)
Rai *et al.* 2002^7^	24	Optional	≤ 17	59	nr	42 (nr)	nr (4)
Fiegl *et al.* 2003^8^ (abs.)	27	nr	nr	nr	nr	nr (nr)	15 (0)
Ferrajoli *et al.* 2003^9^	42	Cotrimoxazole, valacyclovir	≤ 12	35	41	71 (nr)	29 (0)
Stilgenbauer *et al.* 2004^10^ (abs.)	50	nr	nr (sc)	66	34	nr (24)	14 (0)
Moreton *et al.* 2005^11^	91	Cotrimoxazole, acyclovir, g-csf, ganciclovir	≤ 13	48/30	46	43 (24)	15 (0)
Osterborg *et al.* 1997^12^	29	Optional	≤ 3	31	28	93 (17)	nr
Osuji *et al.* 2004^13^ (abs.)	26	nr	nr	nr	nr	nr	17 (4)
Williams *et al.* 2001^28^ (abs.)	1538	nr	nr	nr	nr	nr	3.6 (0.6)
Monotherapy in previously untreated cll							
Hillman *et al.* 2004^14^ (abs.)^d^	297	nr	nr	nr	nr	nr	15^0^
Lundin *et al.* 2002^15^, Karlsson *et al.* 2005^16^ (abs.)	41	Valacyclovir, fluconazole, cotrimoxazole	≤ 2 (sc)	74^e^	16^e^	14 (0)	11 (0)
Combination therapy in relapsed/refractory cll							
Faderl *et al.* 2003^17^	32	Valacyclovir, cotrimoxazole	nr	nr	nr	52 (nr)	27 (0)
Wierda *et al.* 2004^18^ (abs.)	31	Valacyclovir, cotrimoxazole	nr	23/39	23/16	nr (nr)	24 (0)
Elter *et al.* 2005^19^	36	tmp/smx, valacyclovir	≤ 4	26	30	nr (15)	6 (0)
Maintenance/consolidation therapy in patients with cll							
Wendtner *et al.* 2004^20^,^d^	21	Cotrimoxazole, famciclovir	0	64	36	A: nr (64) O: 20 (0)	A: 36 (18) br>O: 0 (0)
Rai *et al.* 2002^22^ (abs.)	56	Acyclovir, cotrimoxazole	nr	nr	nr	nr (33)	22 (0)
O’Brien *et al.* 2003^23^ (abs.)	58	Valacyclovir, cotrimoxazole	0	nr	nr	33 (nr)	21 (0)
Liggett *et al.* 2005^24^ (abs.)	29		F+R: 6 A: 38	F+R: 26 A: 14	F+R: 6 A: 7	nr	nr
Rossi *et al.* 2004^25^ (abs.)	35	nr	nr	nr	nr	34 (nr)	57 (0)

a Percentage of patients experiencing grade 3 or 4 infusion-related toxicities, including constipation, dermatitis, diarrhea, dyspnea, edema, fatigue, fever, headache, hypertension, hypotension, injection site reaction, myalgias, rash, rigour, nausea and vomiting, urticaria.

b Values separated by an oblique (e.g., 48/30) give the percentages of grade 3 and grade 4 events respectively.

c Grade 3 and 4 events may have occurred in this study, but they were not explicitly reported.

d Randomized controlled trials; all other studies are single-arm trials.

e Grades 2 through 4.

cmv = cytomegalovirus; tmp/smx = trimethoprim/sulfamethoxazole; abs. = abstract; g-csf = granulocyte-colony stimulating factor; nr = not reported; sc = subcutaneous administration; F = fludarabine; R = rituximab; A = alemtuzumab; O = observation.
